# Editorial Correspondence of the Peninsular and Independent Medical Journal

**Published:** 1860

**Authors:** 


[From the Peninsular and Independent Medical Journal.

### *Editorial Correspondence of the Peninsular and Independent Medical Journal.*

ABERDEEN, Scotland, Sept. 23d, 1859.

*Dear Readers of the Peninsular and Independent:*

In my last I gave some account of a portion of the medical gentlemen connected with King's College Hospital, and, I think, Middlesex Hospital, London. My design was to go on with the accounts of London men and institutions, until all I had to say of them was completed—then proceeding to describe some of the men and things in Paris, Germany, &c., following the order in which my observations were made—but after passing through Wales and Ireland, I find myself in this distant part of Scotland, in attendance upon the meeting of "*The British Association for the Advancement of Medical Science*," and, under such circumstances of interest as to induce me to deviate from the original design and give in the present letter some account of affairs here—returning afterwards, perhaps, to the original plan. The reasons for deviating in this instance is, that the events, as they are transpiring, will be more fresh in my mind, and will be more interesting to you near the time of their occurrence.

The name of this body is quite explicit as to its great object. It is said to owe its commencement to a discussion which arose, between the years 1826 and 1831, as to the low state of science in England, and the neglect here of scientific men. Sir Humphrey Davy, Sir John Herschel, Sir David Brewster, Professor Playfair, and others of similar character, had expressed their opinions of the superiority of many foreign, to British Scientific Institutions, and their strong feeling regarding the want of encouragement given to scientific men in the country. Sir H. Davy wrote, that in looking back, he found in previous reigns, Boyles, Cavendishes and Howards, who rendered their great names more illustrious by their scientific renown.

"But," he adds, "we may in vain search the aristocracy now for philosophers, and there are very few persons who pursue science with true dignity; it is followed more as connected with objects of profit than those of fame, and there are fifty persons who take out patents for supposed inventions, for one who makes a real discovery."

A Mr. Babbage, Sir David Brewster, and others, wrote in a similar strain, insisting that the higher departments of science had gradually declined since the days of Newton. Sir David concluded an extended article on the subject in the Quarterly Review by saying:

"An association of our nobility, clergy, gentry, and philosophers, can alone draw the attention of the Sovereign and the nation to this blot upon its fame. Our aristocracy will not decline to resume their proud station as the patrons of genius, and our noble names will not renounce their place in the scientific annals of England. The prelates of our National Church will not refuse to promote that knowledge which is the foundation of pure religion, and those noble inquiries which elevate the mind and prepare it for its immortal destination. If this effort fail, we must wait for the revival of better feelings, and deplore our national misfortune in the language of the wise man: 'I returned, and saw under the sun that there is neither bread to the wise, nor yet riches to men of understanding, nor yet favor to men of skill.'"

By appeals like these, and from such sources, in 1831, through the instrumentality of Lord Brougham, the state of science, and its cultivators, was brought before Lord Grey's Government, and some important objects relative to such an association, as had been suggested by Sir David Brewster, were secured. The idea of an association of this kind, though urged with such effect by Sir David—a man who has been described as "a philosopher whose investigations have been extended through almost every branch of physical science,"—was not original with him; for, similar associations had existed in Germany for some years before, and the advantages to each other, of learned men meeting together, had long been understood. The distinguished La Place manifested his appreciation of such meetings when he said:

"The chief advantages of learned societies is the philosophical *spirit* to which they give birth, and which they cannot fail to diffuse over all the various pursuits of the nations among whom they are established. The insulated scholar may, without dread, abandon himself to the spirit of system; he hears the voice of contradiction from afar; but in a learned society, the collision of systematic opinions soon terminates in their common destruction; while the desire of mutual conviction creates among the members a tacit compact to admit nothing but the results of observation, or the conclusions of mathematical reasoning. Accordingly, experience has shown how much these establishments have contributed since their origin to the spread of true philosophy."

The first meeting of the *British Association for the Advancement of Science*, was held at York, in September of 1831, and has consequently been in existence 28 years—the present meeting being its 29th. The first meeting, so efficient had been the efforts to call public attention to the subject, consisted of 353 persons. Lord Milton, then President of the York Philosophical Society, presided, and in his opening address said:

"With regard to the more direct advantages which we have a right to anticipate from these meetings, I have no doubt that, if they shall be extended to different parts of the country, and held in well-selected places, this result will be obtained: the men of science, now scattered over the empire, will be enabled to meet each other, and mutually communicate their ideas; they will state the advances which have been made in their own respective spheres of action, and also what the deficiencies may be. Thus not only will an extraordinary impulse be given, but the individuals and the societies, taking part in the meetings, will learn what part of science they can cultivate with the greatest utility, and will give their researches the most advantageous direction."

Another object of the Association was stated to be, to secure a collection of reports, showing the present state of science, in order that scientific students might know where to begin their labors; and in order that those who pursue one branch of knowledge might know how to communicate with inquiries in another, for it was affirmed that want of such knowledge was constantly found; and also that speculations were published which showed the greatest ignorance of what had been done and written on the same subjects, by others. Besides these general objects, the originators aimed at a repeal or reform of the law of patents, and at direct national encouragement, by government, for science and its cultivators. From this beginning, and with these aims, an account of which I have thought proper to thus somewhat fully present to you, the society has gone on in a course of increasing prosperity and success, and has been a powerful means of raising British science and scientific men to their present proud position. At the first meeting at York, as already stated, 353 tickets were issued—at Oxford, the next year, 564—at Cambridge, in 1833, 856—while at the next meeting at Edinburgh, there were 1,139; and still the next, at Dublin, there were 1,203; and at the meeting at Newcastle, several years after, 2,076 were in attendance—the largest of any until the present meeting, at which nearly 3,000 tickets have already been issued, and many more would have been granted to applicants had the room in which the great meetings are held been large enough to accommodate more. Of the immense numbers in attendance there is a fair sprinkling of ladies—for the most part the wives, sisters, and daughters of members. Of those present between 500 and 600 are full permanent life members, having paid an initiation fee of £10, and after paying the annual sum which entitles them to the volume of Transactions, without farther expense; the remainder being Associate members—paying an annual subscription fee. Special tickets are furnished for ladies at £1 each, entitling them to be present at the meetings; but their names do not appear in the printed lists, as do the regular and Associate members—the two latter classes, however, being in separate pamphlets. There are 26 delegates from different learned societies in Great Britain, and one from the American Medical Association. There are a considerable number of gentlemen present, interested in science from foreign countries, representing France, Holland, Germany, Austria, Russia, and the United States.

Though the Association is steadily gaining in favor with scientific men and the country, yet much of the *eclat* of the present meeting has been given by the fact of the Prince Consort acting as President—besides the curiosity existing everywhere of seeing personages of high position, there is among most of the British people a peculiar admiration for rank and title, and a strong feeling of loyalty indulged without restraint towards their present worthy *sovereign*, which makes them almost worship—at least show the greatest interest in every one connected with the Royal Family. Besides all this, Prince Albert is, from his unexceptionable character, and his manifest interest in science and improvement, personally popular; and all these circumstances have doubtless contributed to bring together many who otherwise would have pursued other objects of interest or amusement.

The Association convened on the 14th Sept. inst.—the *Council* at 10, A. M., the *General Committee* (which does most of the arranging of affairs and general business) at 1, P. M., the *General Meeting* occurring at 8½, P. M.; when the retiring President, Prof. Owen, yielded the chair to his successor in a brief speech, and Prince Albert proceeded to deliver an opening address. It was an unpretending, but clear and sensible production, and was well adapted to the occasion. He spoke in becoming terms of his inferiority as a man of science, compared with others around him, and claimed indulgence in his attempts to perform his duties, especially so, as he succeeded to "a man of whom the country was justly proud, and whose name stood among the foremost of the naturalists in Europe, for his patience in investigation, conscientiousness in observation, boldness of imagination, and acuteness in reasoning."

In mentioning the reasons which determined him to accept the office, he said:

"Remembering that this Association is a popular Association, not a secret confraternity of men jealously guarding the mysteries of their profession, but inviting the uninitiated, the public at large, to join them; having as one of its objects to break down one of those imaginary and hurtful barriers which exist between men of science and so-called men of practice—I felt that I could, from the peculiar position in which Providence has placed me in this country, appear as the representative of that large public which profits by and admires your exertions, but is unable actively to join in them; that my election was an act of humility on your part which to reject would have looked like false humility, that is like pride on mine. But I reflected further, and saw in my acceptance the means, of which necessarily so few are offered to her Majesty, of testifying to you, through the instrumentality of her husband, that your labors are not unappreciated by your Sovereign, and that she wishes her people to know this as well as yourselves."

He then spoke of the objects of the Association in very proper terms, and proceeded to give his notions of science; the following extract serving as a specimen of his style of thought and expression, and being worthy of being reproduced:

"To me, science, in its most general and comprehensive acceptation, means the knowledge of what I know, the consciousness of human knowledge. Hence, to know is the object of all science; and all special knowledge, if brought to our consciousness in its separate distinctiveness of form, and yet in its recognized relation to the totality of our knowledge is, scientific knowledge. We require, then, for science—that is to say, for the acquisition of scientific knowledge—those two activities of our minds which are necessary for the acquisition of *any* knowledge—analysis and synthesis; the first to dissect and reduce into its component parts the object to be investigated, and to render an accurate account to ourselves of the nature and qualities of these parts by observation; the second to recompense the observed and understood parts into a unity in our consciousness, exactly answering to the object of our investigation. The labors of the man of science are therefore at once the most humble and the loftiest which man can undertake. He only does what every little child does from its first awakening into life, and must do every moment of its existence; and yet he aims at the gradual approximation to divine truth itself. If, then, there exists no difference between the work of the man of science and that of the merest child, what constitutes the distinction? Merely the conscious self-determination. The child observes what accident brings before it, and unconsciously forms its notion of it; the so-called practical man observes what his special work forces upon him, and he forms his notions upon it with reference to this particular work. The man of science observes what he intends to observe, and knows why he intends it. The value which a peculiar object has in his eyes is not determined by accident, nor by an external cause, such as the mere connection with work to be performed, but by the place which he knows this object to hold in the general universe of knowledge, by the relation which it bears to other parts of that general knowledge.

"To *arrange* and *classify* that universe of knowledge becomes therefore the first, and perhaps the most important, object and duty of science. It is only when brought into a system, by separating the incongruous, and combining those elements in which we have been enabled to discover the internal connection which the Almighty has implanted in them; that we can hope to grapple with the boundlessness of His creation, and with the laws which govern both mind and matter. The operation of science, then, has been systematically to divide human knowledge, and raise, as it were, the separate groups of subjects for scientific consideration into different and distinct sciences."

He spoke for about fifty minutes with a fairly distinct and agreeable voice, and with a moderate German accent, and his effort was very well received by one of the most intelligent audiences ever assembled, and which seemed fully to sympathize with Sir Benjamin Brodie, who, in moving a vote of thanks, said:

"Gentlemen of the British Association!—His Royal Highness has performed the task which he had undertaken, and I think we have a duty to perform before this meeting separates. Of the address His Royal highness has just delivered, I might say much more, if I were not prevented by the presence of His Royal highness; but this much I may venture to say, that we may trace in it the same signs of strong and sound sense, clearness of comprehension, varied knowledge, and good intentions, which are to be found in all the addresses which His Royal Highness ever made in public. [Applause.] I am sure that we must all feel that this country is greatly indebted to His Royal Highness for the attention which he pays, on all occasions, to the advancement of knowledge and improvement of our social system, and for contributing as he does in every way to promote the happiness of this his adopted country. [Applause.] I am sure you will all agree with me that the we ought to express our cordial thanks to His Royal Highness, for the address which he has just delivered, for having consented not only to adorn this meeting with his presence, but to take the chair and make such exertions for us. [Applause.] I am sure you will all join with me in hoping that he may long be spared to be an honor and a credit to this country. [Great cheering.]"

The scientific work of the Association, which, after all, is its great feature, is done in sections, eight in number, holding their sessions at the same time in different rooms, from 11 o'clock to 3. These are as follows: of Mathematical and Physical Science—presided over by the Earl of Rosse, F. R. S.; of Chemical Science—President, Dr. Lyon Playfair, C. B., F. R. S.; of Geology—President, Sir Charles Lyell, L. L. D., D. C. L., F. R. S.; of Zoology and Botany—President, Sir W. Jardine, Bart., F. R. S. E.; of Physiology—President, Prof. Sharpey, F. R. S.; of Geography and Ethnology—President, Admiral Sir J. C. Ross, D. C. L., F. R. S.; of Economic Science and Statistics—President, Col. Sykes, F. R. S., M. P.; and of Mechanical Science—whose President is the Rev. Prof. Willis, F. R. S., of the University of Cambridge.

In these eight sections, during their six working days now just brought to a close, 371 papers were read, (generally in full, though sometimes by abstracts,) and discussed. This enormous number embraces almost every variety of scientific subject, from the minutest molecule and the laws which govern its being, to the largest bodies, and the most momentous interests in the universe, and the great principles connected with their existence and operations. Not only matters of physical, chemical, and vital science were discussed, but Economics and Statistics were brought under consideration, and some of the most able and animated discussions of the meeting were held in this section. Such subjects as the following: Statistics of the Trade and Progress of the Colony of Victoria, Australia; statistics of the Small Pox and Vaccination in the United Kingdom; statistics of the Free Church Building in Glasgow; Trade and Commerce of India; the Industrial Feeding Schools of Aberdeen; the effect of the Influx of the Precious Metals which followed the Discovery of America; the Social and Economical Influence of the New Gold of California and Australia; Statistics of Color Blindness; Decimal Coinage; Progress of Public Opinion with respect to the evils produced by the traffic in Intoxicating Drinks, as at present regulated by law, &c.—were ably reported upon and elicited a large amount of interest.

The division in which I took most interest, and which I attended most constantly, was that of Physiology, embracing as it did, in its various papers and the discussions upon them, many pathological subjects as well. The following are the subjects of the principal papers produced:

Professor Bennett—On the Structure of the Nerve Tubes.

Dr. Redfern—On the Admixture of Nervous and Muscular Fibres in the nerves of the Leech.

Bernard E. Brodhurst, F. R. C. S.—On the Repair of Tendons after their Sub-Cutaneous Division.

Dr. Foster—On the action of the Heart of the Snail.

G. H. Lewes—On Improved Processes of Physiological Investigation.

G. H. Lewes—On the Supposed Distinction between Sensory and Motor Nerves.

John Adamson, M. D.—Case of Lactation in an unimpregnated Female of the Canis Familiaris.

Professor Allman—Report on the Reproductive Organs of the Hydroid Zoophytes.

George Ogilvie, M. D.—The Genetic Cycle in Organic Nature.

Professor Laycock—Handwriting and Drawings of the Insane, as illustrative of some modes of Cerebral Functions.

Professor Bennett—On the Origin of Morbid Growths, with reference to the Connective Tissue Theory.

John Duguid Milne, Jr., M. A.—On the Homologous Development of the Muscular System.

Robert Garner, F. L. S.—Reproduction in Gasteropoda, and on some curious effects in Endosmosia.

John Marcel, M. D., F. R. S.—An Experimental Inquiry into the action of Alcohol on the Nervous System.

Professor Bennett—On the Molecular Theory of Organization.

W. E. C. Nourse, F. R. C. S.—On the Organs of the Senses, and on the Mental Perceptive Faculties.

A. B. Garrod, M. D., F. R. S.—On the Specific, Chemical and Microscopical Phenomena of Gouty Inflammation.

G. H. Lewes—A Demonstration of the Muscular Sense.

George Rainey, M. R. C. S.—On the Structure and Mode of Formation of Starch Granules, according to the principle of Molecvlar Coalescence.

John Dennis Macdonald, R. N., F. R. S.—On the Homologies of the Coats of Tunicata, with remarks on the Physiology of the Pallial Sinus System of Brachipoda.

Professor W. W. Fisher—Illustrations of the Normal Development of the Vertebrate System, by its Abnormal States.

Edward Smith, M. D., L. L. D.—On the Sequence observed in the Phenomena observed in Man under the Influence of Alcohol.

Alphonse Gages, M. R. S. A.—On the Comparative Action of Hydrocyanic Acid on Albumen and Caseine.

Richard Fowler, M. D., F. R. S.—A Second Physiological attempt to unravel the perplexities of the Hypothesis of Berkley.

William Camps, M. D.—On certain imperfectly recognized functions of the Optic Thalami.

William Camps, M. D.—On Certain Subjective Sensations, with especial reference to the Phenomena of Second Sight, Visions and Apparitions.

A brief account of some of these papers and their authors, will constitute the burthen of the remainder of this letter, already becoming long.

The author of the first paper on the list, Prof. Bennett, of

Edinburgh, is the same who has recently given to the world a large work on "*Clinical Medicine*," and who has distinguished himself by so strenuous an advocacy of abstinence from blood-letting, and other depleting measures, in the treatment of Inflammations. I was curious to see this gentleman, and to get an impression of the character of his mind, as evinced by his appearance and efforts, otherwise than upon paper; and have had an excellent opportunity in his frequent participations in the discussions of the section of the Association in which he takes a most active part. He is a man rather less, than above, fifty, scarcely looks older than forty, of about medium size, with a short nose, and not an expanded brow. He has an active, vigorous temperament, and is evidently fond of controversy and disputation; but I must say, that what I have seen of him has not strongly impressed me with the clearness of his impressions, the earnestness and depth of his convictions, or the rigor of his logic. I do not intend to say that he is markedly deficient in any of these respects, but, simply that, in my judgment, he is not eminent in either. That he has a good amount of talent—that he is active and industrious, his numerous productions prove. That he has the ability to inspire others with zeal and activity in the scientific and professional pursuits, his students testify—but that he is one of those clear-headed, unprejudiced men, whose enlarged views, expanded powers, and unquestionable purposes, render them eminent as authorities, cannot be fairly claimed; and, although a useful contributor to science, his conclusions,) as should in fact be the case with every man's,) must by no means be received without the most rigid investigation. I feel in duty bound to say at least this much, because it is my strong conviction, and because his works and opinions are becoming extensively disseminated throughout our country, and many of them, in my judgment, are far from being sustained by the largest observation of facts, and the most careful inductions of reason. These remarks are not made with special reference to his papers presented to the section of the Association, with which you will be less interested with the question,—whether, when you have a strong, well-nourished patient, with a full muscular system, attacked with a sthenic inflammation, you shall not draw blood as a powerful means of diminishing that action, and preventing its development into all the consequences which may follow? It is to enable you to give Dr. B.'s authority its proper weight on this and other great practical questions, rather than those of a more abstruse character respecting the ultimate structure of the nerve-tube, or even the great molecular theory of organization that I have made these remarks.

The chief idea in the first paper is that the fluid in the nerve-tube is capable of being coagulated by certain manipulations and re-agents, and that the curious structures observed by various observers and authors, do not exist in the semi-fluid or central part of the tube, but are the results of these chemical changes—of a partial coagulation. He thinks the nerves are simple tubes, containing a viscus fluid, of unknown, but in different parts, various chemical composition—hence, being variously effected by re-agents in the different parts, producing the central, flatten ed, and waving fibre, and all the other forms observed by Clark, and others, but that the *Structure* is uniform.

Prof. Allen Thomson, of Glasgow, agreed with Prof. Bennett, in much he had said, but thought there was a difference of structure as well as of chemical composition in the interior of nerve-tubes—that there is a medullary sheath and a central body. However, he thought nothing was very positively known on the subject, and it was hardly proper to come to conclusions on the data given.

Prof. Sharpey, President of the Section, and a noble specimen of a clear-headed, honest-hearted, scientific man—large and erect in body and mind, said: even Dr. Bennett did not deny that there was at least a difference of consistence in the different portions of the contents of the nerve-tube, and he thought this involved something more. The central axis or central filament, as described, differed in structure from the rest of the contents of the nerve membrane or tube. The different parts were more or less independent of each other. He had watched the growth of nerves, and found that the central parts of filaments grow first. The ultimate nerve tapers at its extremity, when growing. Still he admitted that many of the changes described by authors were probably mere fibrilation occurring after death, or in consequence of re-agents.

Dr. Acland, formerly Professor of Anatomy, now of Medicine, at Oxford University, thought it was dangerous to truth to draw conclusions as speedily and decidedly as some did on the structure of the nerves. Many of the appearances were doubtless produced by manipulations and re-agents, and it was difficult to say how far these differences, when produced, indicated original difference of structure. But there was a difference between the external and internal membrane of the nerve-tube, and the central axis differed from the rest of the contents. This difference was chemical or structural, or both—quite likely both; but we should be careful about attaching difference of functions to these different parts. Our knowledge was not sufficiently advanced for that.

Dr. Redfern, Professor of Anatomy and Physiology in the University of Aberdeen, said: True Physiologists are very cautious in these respects. They gave only what they saw; and, in these cases under discussion, observations were not so perfect as to entitle one to draw physiological conclusions. He requested information as to the difference between sympathetic and common nerve-tubes.

Dr. Sharpey said:

This question, like many others, was more easily asked than answered.

Dr. Bennett said:

He did not deny there were differences of some kind in different parts of the nerve-tube or fibre. The central portion, or as it had been called, band, had more affinity for coloring matter, for instance; but the question was as to structural difference. He could not see it in the recent state.

Dr. Acland said:

Dr. Bennett, after all, proved there was a difference, (he seemed to start with the position that there was none), slight, perhaps, but yet a difference, as shown by the effects of water and other agents. A slight difference in structure, or even in chemical composition, may cause a decided difference in function; so that he scarcely saw what Dr. Bennett's paper had made out. Here I find in my notes (these sketches are from notes taken at the time of the reading of the papers, and the discussion on them,) these inquiries: May not chemical differences in the different portions of a nerve modify the functions as much as structural differences? Is it not indeed probable that chemical affinity has much to do in conveying impressions through nerves?

Dr. Redfern's paper, the next on the list, merely affirmed that he had seen, some years ago, movements in the nerve of a leech carefully dissected out for a considerable distance from its surrounding connections. The inference was that nerve substance had the power of spontaneous oscillatory movements. He had made many inquiries on the subject without obtaining light, but had recently examined the leech carefully, and had found muscular fibres in the sheath, and combined with the sympathetic nerves. When nerves are cut off from other connections, but left with their ganglia, some will be found to bend in a rainbow form. Those that do so will be found to have muscular fibres attached to them within their sheaths, and on the side towards which the curve takes place. His observations have been made on leeches, but he has no doubt the same will be found true with regard to other of the lower classes of animals.

The author of the next paper, Mr. Brodhurst, is one of the Assistant Surgeons to the Royal Orthopœdic Hospital, London, and, though a young man, has had opportunities for practical observation in the many cases of subcutaneous divisions of tendons occurring there. His paper was of much practical interest to surgeons, but abounded in details too lengthy to be recorded in this letter. I shall attempt to give only a few of the conclusions.

When a tendon has been divided, its ends should be approximated, or union may not take place. The distance varies at which union may occur in the divided ends, but there are limits in all cases, and in some the distance must be small. After union has taken place, the new connecting material may be extended very much by mechanical means, while it is new. It then becomes permanent structure in the extended form. Motion, as exertions of the muscles attached, will sometimes prevent union when the cut ends are not far asunder. If the tendons, after being cut, are separated more than two inches, union is not likely to occur. The formation process commences at the cut ends of the tendon, and is built out in that way; but the sheath of the tendon is useful in giving it form. In the case of long ligamentous substance between the fragments of a broken patella, it is probable that union occurred when the bones were approximated, and the new substance extended afterwards by the action of the strong muscles. When much force is applied to the new tendon when recent, it may be greatly elongated—several inches —so as even to render the muscle with which it is attached powerless. While the new tendon is recent, if all tension is removed from it, contraction is apt to occur. After a time, as a few months, it becomes fixed and permanent as other structures.

Sir Benjamin Brodie and Prof. Sharpey asked the reporter several questions which elicited a portion of the facts above recorded.

Dr. Foster's paper, the next in order, tended to show that the heart of snails, when cut into small pieces, continued to beat for a considerable time, each piece beating rythmically—wherefrom the inference was drawn, that the heart of these animals had an inherent power of rythmical contraction within itself, independent of nerves. In a somewhat lengthy discussion which followed on this paper, Dr. Bennett agreed with the reporter generally.

Prof. Huxley, of London, said these experiments applied to the frog would have met with different results. In the higher animals, the heart is evidently under the controling influence of nerves.

Prof. Sharpey suggested that there might be small ganglia in the heart tissue, causing each part in which the ganglia was situated to beat under proper stimuli independently; but yet the different particles influenced others near them. Something of this kind was found in the intestines. He compared this arrangement to a team of horses—each had a motion from its own volition, but one horse was influenced by the others, and all were governed by the driver, with his reins and whip. The latter he compared to the nerves which usually governed and harmonized all such motions.

Profs. Thomson and Huxley favored the idea of the inherent motive power of muscles, independent of nerves, coming back to the old views of Haller.

Sir B. Brodie said:

Spermatoza acted rythmically though they had no nerves. Yet nerves do unquestionably influence the heart.

Dr. Bennett said :

The heart of a chick beat before it had any definite structure —while there were nothing but cells apparent.

But, said Dr. Thomson, this is an incipient heart with all its parts in prospect.

For my own part, I cannot confide in the observations of the reporter as to the fact. It seemed to me, from the manner of his observations, that he might have been mistaken. The movements were seen several minutes apart, and it occurred to me there was an opportunity for self-deception.

Mr. G. H. Lewes—not a medical man but an able popular writer—sent in three papers on physiological subjects, the titles of which are in the list. He attacked prevailing views with boldness and vigor, and brought some out severely against him, especially Dr. Bennett.

Mr. L. found fault with a want of accurate definitions of *sensation*, and various other terms used. Announced as a trueism that identity of structure gives identity of function, and diversity of structure diversity of function ; declared the structure of what we called sensory and motor-nerves to be identical—questioning the common distinction ; declared the gray matter of the nerves to be identical with the gray matter of the brain ; inferred identity of function—a force of their own, like brain force ; proposed new terms—neurility and sensibility, giving each specific meanings, &c., &c. Profs. Sharpey, Thomson and Huxley, and Sir B. Brodie, were more liberal and fair in their remarks, admitting, as all must, that we have much in relation to the physiology of the nerves to improve and learn. The discussion was exceedingly interesting, but took too broad a range, and was too much diversified to be reported here.

For my own part, I felt much interested in Mr. Lewes, who was not present, and in a subsequent discussion upon another of his papers, defended some of his positions, which I believed to be correct. I hope he will go on with his physiological writings. It is alleged that he is an " outsider," but outsiders, in regard to any science or subject, may be useful critics, nevertheless. We often consult even children as to the faithfulness of a picture to nature, and profit by their opinions. Improvements in medical science have sometimes come from non-professional persons ; political improvements from those who have not been educated in politics. But Mr. Lewes, though not a professional man, has studied, it was stated, physiology thoroughly—was well informed of its literature—perhaps much better than some who might criticise him. While I would not receive such a man as an authority, I would listen to him as a witness, and would receive his suggestions for what they were worth.

I have thus given you a brief and exceedingly imperfect report of a single day's proceedings, in one of the eight sections of the British Association for the Advancement of Science, and this, with other matters mentioned, will enable you, I hope, to form some notion of its character. I should, however, further say that two of the evenings since the body has been in session, besides the opening, have been occupied with lectures in the large Musical Hall, before the whole body. One by Sir R. J. Murchison, Director General of the Geological Survey of the United Kingdom, "On the Geology of the Northern Highlands of Scotland," another by the Rev. Dr. Robinson, Director of the Armagh Observatory, "On Electrical Discharges in highly Rarified Media;" and two other evenings were spent in general conversaziones in the Music Hall and rooms adjoining, where a large number of objects of scientific and historic interest were exhibited, and where promenades and refreshments were enjoyed—coffee, tea, &c., but no wines were provided. Large numbers of ladies were present on these occasions. The Geological lecture was illustrated by large drawings, and the Electrical by the most brilliant experiments of the kind I have ever witnessed. During the electrical discharges into these Rarified Media, the Hall was darkened, and the most beautiful colored lights produced of which it is possible to conceive.

The concluding meeting—a general one—took place on Wednesday, the 21st, at 3 o'clock, P. M., when a report was made of the proceedings of the General Committee, showing an appropriation of £930 for the coming year; for various scientific enquiries, &c., and various congratulatory and laudatory speeches were made.

During the session of the Association, a note was received by the Secretary from the Prince Consort, inviting, in the name of Her Majesty, the members of the General Committee, including foreigners in attendance, to a breakfast on Thursday at Balmoral, her Scottish residence, about fifty miles from Aberdeen, among the Highlands. It was, of course, accepted, and at 6 o'clock in the morning of that day, 200 persons, including most of the distinguished men of the Association, were on their way, seventeen miles being of railway and the remaining thirty-three by omnibusses. This route was a most beautiful one, up the river Dee, presenting many characteristic scenes of the Highlands, bringing us to the Palace about 2 o'clock.

This letter is already too long, and I shall attempt no detailed description of our entertainment. A good deal of preparation had evidently been made for our reception and enjoyment. There was a large collection of people within the grounds, tents were pitched about, bands of music, companies of Highlanders in their national costumes, were present, and during the afternoon a large number of the Scottish national games, such as throwing a huge sledge, carrying and throwing over an immense pole, foot racing, and dancing the "Highland Fling," were performed; the Queen giving prizes to those who excelled in these muscular feats. The Queen, and her husband and family, were dressed in Scottish costume, and entirely unattended by any guards—not a musket was in sight—was surrounded by her guests, and almost literally mingling with the people around. She is a bright, energetic, kind-looking little woman, and the Prince of Wales and the rest of the family are very pleasant, good-looking children. They all seemed in excellent health, and under excellent physical, mental, and moral discipline. Both the Queen and her husband appear remarkably young for persons of their age, and in no way appear to differ from other well-dressed, well-behaved, respectable people. Looking with purely republican eyes, it seems strange that so much interest is concentrated upon one little woman; innocent, sensible and amiable though she be, but the sentiment of loyalty is strongly rooted in the English, and indeed, in the Scottish heart, and in the present state of the world it may be well that it is so. There could certainly be no more unexceptionable Royalty than that which now amuses and interests the British nation; and, as I left the royal presence and mansion, with the effects of royal good cheer upon me, I could but join in the general wish for the good health and long life of Her Majesty, and as the bands played "God save the Queen," a fervent, heart-felt response could but arise.

Yours, truly, A. B. P.

